# Skin Cancer Prevalence in Outdoor Workers of Ski Resorts

**DOI:** 10.1155/2020/8128717

**Published:** 2020-01-28

**Authors:** Yolanda Gilaberte, Josep Manel Casanova, Ana Julia García-Malinis, Salvador Arias-Santiago, Maria Reyes García de la Fuente, Marta Pamiés-Gracia, Javier Ramirez-Palomino, Isabel Ruiz-Campos, Tamara Gracia-Cazaña, Agustín Buendia-Eisman

**Affiliations:** ^1^Department of Dermatology, University Hospital Miguel Servet, IIS Aragón, Zaragoza, Spain; ^2^Department of Dermatology, University Hospital Arnau de Vilanova, Lleida, Spain; ^3^Unit of Dermatology, Hospital San Jorge, Huesca, Spain; ^4^Medical School, Granada University and Department of Dermatology, Hospital Virgen de las Nieves, Granada, Spain; ^5^Vielha Hospital, Vielha (Lleida), Spain; ^6^Medical School, Granada University, Granada, Spain; ^7^Unit of Dermatology, Hospital de Barbastro, Barbastro, Spain

## Abstract

**Background:**

Snow reflectivity and altitude increase the exposure of ski resort workers to solar ultraviolet radiation. The aim was to assess the presence of skin cancer in ski resorts workers and compare it with other groups of outdoor workers reviewing published studies.

**Methods:**

An observational cross-sectional prospective study was conducted in the three largest ski resorts in Spain: Baqueira Beret, Lleida; Formigal, Huesca and Sierra Nevada, Granada. All outdoor workers including ski instructors were invited to participate in the study. The participants completed a validated questionnaire about sun exposure and underwent a skin examination.

**Results:**

219 workers were included in the study (80% male; mean age 43.8 (SD 11.31) years). Actinic keratosis (AK) but no other skin cancers were detected in 32 participants (14.62%). Those with AK worked in the Southernmost ski resort, were more likely to have light colour hair, and were older and with higher photoaging grade than those without them.

**Conclusion:**

Compared to other studies, outdoor workers on ski resorts show a higher prevalence of AK than general population but a lower prevalence than other groups of outdoor workers.

## 1. Introduction

The harmful effects of ultraviolet radiation (UVR) on skin have been known for several decades. Long term exposure is the primary risk factor for photoaging [1] and skin cancer, besides UVR has been classified as a human carcinogen [2]. A high level of sun exposure is a key feature of outdoor work. Two meta-analyses have provided epidemiological evidence that outdoor workers have a significantly higher risk of developing nonmelanocytic skin tumours (basal cell and squamous cell carcinomas) than indoor workers [3, 4].

In mountainous areas levels of ambient UVR are increased as a consequence of the combined effects of a shorter radiation pathlength, a low aerosol load, and the reflectivity of snow [5, 6]. The UVR levels to which ski instructors and mountain guides in the Alps are exposed exceed the threshold of 80 J.cm^2^ per 8 hours of work, calculated using the CIE reference spectrum (1987) normalized at 298 nm, by 9–53 fold [7]. Moreover, studies conducted in several countries have concluded that in ski-fields the UV irradiance on horizontal surfaces is 20–30% higher than that recorded at sea-level sites, and personal radiation doses are consistently higher than those received on beaches at similar latitudes [6]. Spain is one of the most southern countries in Europe and home to many ski resorts, including Sierra Nevada, the most southerly ski resort in Europe. The ultraviolet index in these resorts are even higher than those recorded in other ski resorts in Europe; in February the average of UVI in Spain is 3 against France and Germany which is 1 [8]. In this study, we sought to quantify clinical signs of chronic sun damage, the prevalence of skin cancer and photoaging severity, in outdoor workers in Spanish ski resorts. Additionally, we assessed knowledge and habits relating to photoprotection in this population, and finally compare in the discussion the results with those published to date in the literature on other risk groups of outdoor workers.

## 2. Material and Methods

### 2.1. Design

This observational cross-sectional prospective study was conducted in the three largest ski resorts in Spain: Baqueira Beret, Lleida (42°41′N, 0°56′E, altitude 1500 to 2610 m); Formigal, Huesca (42°45′N 0°23′O, altitude 1550 to 2250 m); and Sierra Nevada, Granada (37°05′N 3°23′O, altitude 2100 to 3300 m).

### 2.2. Study Population

All outdoor workers including ski instructors were invited to participate in the study. After providing written informed consent, all participants underwent an interview and a physical examination carried out by a dermatologist at the medical facility of their ski resort. Data were collected at the end of the working day over several days at the end of March and beginning of April, 2016.

For inclusion in the study participants were required to fulfil the following criteria: to work outdoors in any of the three ski resorts, and to be exposed to sun for at least 4 hours per day, 3 days per week, for at least 11 weeks between the end of November 2015 and mid-April 2016. Indoor workers and those that did not provide informed consent were excluded from the study.

### 2.3. Questionnaire and Physical Examination

Sun exposure behaviors and knowledge about photoprotection were assessed using the validated Beach Questionnaire, adapted to a snowy environment [9].

The questionnaire was validated in Spain in 422 respondents, in terms of attitudes toward sun exposure, sunburns, sun protection behaviors and knowledge related to sun exposure displayed high degrees of reproducibility in the test–retest procedure [9].

After completing the questionnaire, all participants were interviewed by any of the 6 dermatologists of the study who collected data on their family and personal history of skin cancer and performed a full-body dermoscopy examination, recording the following information: eye and hair colour, phototype, photoaging grade according to the Glogau scale (type 1, no wrinkles; type 2, wrinkles in motion; type 3, wrinkles at rest; type 4, only wrinkles [10]), number of actinic keratosis (AK) lesions and corresponding Olsen grade; number and location of basal cell carcinoma, squamous cell carcinoma, or melanoma lesion and also of melanocytic nevi and atypical nevi; presence of solar erythema (sunburn); presence of freckles on the face or upper back (absent, few, some, many). On completion of the clinical examination the participants were orally informed by the dermatologist about risk factors, any suspicious lesions, and photoprotective measures.

In addition, a DSM II skin colour meter (Cortex Technology, Denmark) was used to measure skin melanin index and erythema on the skin of the cheek and the armpit to evaluate differences between a chronically photoexposed area and a nonphotoexposed area. The definitive value was calculated as the mean of 4 separate measurements taken in each area.

### 2.4. Statistical Analyses

All variables were analysed using descriptive analysis. Qualitative variables are presented as proportions and quantitative variables as measures of central tendency (mean or median) and dispersion (standard deviation or percentiles), depending on the results of the Kolmogorov-Smirnov test.

Associations between qualitative variables were assessed using Pearson's chi-squared or Fisher's exact test. Parametric or nonparametric statistical tests were used as appropriate after assessing data normality. Logistic regression was used to determine those variates associated to the presence of AK. Crude odds ratios (OR) and 95% confident intervals 95% (CI 95%) were estimated. The threshold for statistical significance was set at *p* < 0.05. Analyses were conducted using SPSS version 19.0 (IBM, Armonk, NY).

## 3. Results

### 3.1. Study Population: Demographic Characteristics and Skin Cancer Risk Factors

Table in supplementary material shows the characteristics of the study population. In total, 250 workers participated in the study, but only 219 were included in the analysis (31 were excluded due to the absence of questionnaire or interview data): 60 from Sierra Nevada (27.40%); 71 from Baqueira (32.42%); and 88 from Formigal (40.18%). Males accounted for 80.8% of the population, and the mean (± SD) age was 43.8 ± 11.31 years (range, 19–66). The mean duration working on ski resorts was 20.21 ± 12.92 years (range, 1–50). The most common self-reported phototypes were II (34.7%), III (29.2%), and IV (17.4%). Dark brown hair (49.6%) and brown eyes (33.3% dark and 26.9% light) predominated among the participants. Only 4 participants had a personal history of basal cell carcinoma, 3 of actinic keratosis, and 1 of another form of skin cancer.

Most participants had less than 10 nevi on the back (56.6%), the anterior part of the trunk (71.2%), and the upper extremities (70.3%), being the lower extremities the area with fewer number (92.7%). Few participants had atypical nevi: the most frequent location was the trunk (8.7%), followed by the upper extremities (2.8%), lower extremities (1.8%), and the head and neck (1.4%).

Evaluation of photoaging using the Glogau score revealed the following distribution: I, 16.9%; II, 38%; III, 36.1%; and IV, 9.1%.

Table 1 shows the use of sun protection measures. Sunglasses was the photoprotection measure most commonly used (99.1%) followed by hats or helmets by 93.6% of the sample. Seventy-two percent always used sunscreen with a SPF ≥ 30 and 20.1% used it sometimes, although 59.8% applied it only once a day. Shade was the least used photoprotection measure.

### 3.2. Prevalence of Skin Cancer Lesions

AK lesions were detected in 32 participants (14.62%). Most lesions were solitary with an Olsen grade of I or II. Of workers with AK lesions, all were men, with a mean age of 51.7 ± 20.09 (range, 35–66) years and mean of 29.09 ± 9.75 (range, 10–50) years working in ski resorts. Two had personal history of basal cell carcinoma and another 2 of AK.

Patients with AK lesions were significantly older, had been working for longer in ski resorts (29.09 ± 9.75 vs. 18.35 ± 12.85 years, *p* < 0.001) and have higher photoaging grade (Table 2). Blond or red hair (*p* = 0.039), >10 nevi on the back (*p* = 0.039), and freckles on the back (*p* = 0.021) were also positively associated with the presence of AK lesions. Of the photoprotective measures analysed, the only one for which a significant association with presence of AK was observed was the use of shade, higher by those with AK (*p* = 0.044) (Table 3). No statistically significant differences in the erythema or the melanin index were observed between participants with AK lesions and those without them; however, the mean of the melanin difference between the face and the armpit was higher in those without AK (9.75, SD 9.26) than in those with AK (6.40, SD 7.73) (*p* = 0.06) (Table 4). All the variables for which the univariate analysis revealed a significant association with the presence of AK were included in the multivariate analysis. Cualitative variates with more than two categories were categorized in two as follows: ski resorts (Sierra Nevada in the south vs Formigal and Baqueira Beret in the north); marital status (being married, divorced or viedower vs being single); hair colour (black and dark brown vs light brown, blonde or red hair); photoaging (3 and 4 grades vs 1 and 2 of Glogau); number of nevi on the back (more than 25 vs 25 or less); freckles on the back (moderate and high vs absent of few); use or shade and photoexposed between 11:00 to 16:00 h (sometimes and always vs never). Logistic regression identified the following factors as predictors of having AK: the southern ski resort (OR = 27.54, CI95% 6.89–110); photoaging grade (OR=14.32, CI95% 3.50–58.62); age (OR = 1.10, CI95% 1.05–1.94); and light hair colour (OR = 4.7, CI95% 1.55–14.46) (Nagelkerke R2 = 0.556 without significant differences in the Hosmer-Lemeshow test results (*p* = 0.772).

## 4. Discussion

This analysis of a population of outdoor workers in ski resorts reveals the presence of AK, but no other skin cancers. This presence was associated with the latitude of the ski resort, light hair, and correlated with age and photoaging grade.

The prevalence of nonmelanoma skin cancer (NMSC), including AK, was lower in our study population (14.62%) than that reported in mountain guides (33.3%) and farmers (27.4%) in Germany [11]. In fact, Lichte et al. found a prevalence of more than 7% BCC, 1.4% SCC, and 1 melanoma in a population of 309 mountain guides in Germany [12]. Zink et al. [13], in a group of 62 ski guides from southern Germany (55 men and 7 women; mean age 52.9 ± 13.4 years, range 30–78), reported a prevalence of AK (35.4%) and diagnosed NMSC in 8.1% of participants. In our population, in all cases AK lesions were located on the face, whereas Zink and colleagues reported the presence of 2 BCCs on the upper back and other lesions on the lower legs and lower arms. It should also be noted that only 16.1% and 43.5% of the ski guides in the Zink's study reported wearing long-sleeved shirts and long trousers, respectively (the legs and arms of our participants were covered at all times). Another possible factor is that whereas mountain guides work all year long, ski workers in Spain only work from December to April.

Compared with the Epiqa study of a Spanish dermatology outpatient population (aged ≥45 years) [14], the prevalence of AK in our population was double for participants aged 45–50 years (34.4% vs. 12.6%), and was also higher for those aged 51–60 years (34.4% vs. 26.2%). Even in those under 45 years the prevalence in our group was 15.6%.

Working in the ski resort located in the South of Spain vs those in the North, older age, higher Glogau grade, and light hair color were significantly associated with the presence of AK in our study. These results partially agree with those reported by Flohil et al. [15] in the Rotterdam Study and Fargnoli et al. [16] in Italy who found a significant association of AKs with male gender, light pigmentation status (based on eye, hair, and skin color), skin wrinkling, older age, and having between 25–50 nevi and facial solar lentigos, among others. However, accoding to the multivariate analysis, the most relevant factor was the place of the ski resort, something which agrees with the fact that keratinocyte cancer incidence is directly correlated with latitude, either in general population or in UV-exposed workers [17].

In our study, phototype was not a determinant of AK. However, the mean of the melanin differences between the face and the armpit was higher in those without AK. Our findings support those of a previous study in which the melanin index of unexposed skin was proposed as a precise method for quantification of the risk of sun damage better than Fitzpatrick skin phototype [18].

Regarding the photoprotection measures, sunglasses, hats, or helmets and high SPF sunscreens are usually used by the high majority of our ski workers. They show better sun protection habits than those reported by Buller et al. in adult skiers, with 49.8% wearing sunscreen with SPF 15 or higher and only 20.4% reapplying it [19]. Only the higher use of shade was significantly associated with a higher presence of AKs. There are not studies that evaluate the preventive effect against NMSC of solar protection measures different from sunscreen [20]; although a beach umbrella alone (only shade) did not provide sufficient protection for extended UV exposure compared to sunscreen [21].

A limitation of the present study is the absence of a control group with which to compare our findings. The influence of the occupation (outdoor or indoor) outside of the time at the ski resort was not evaluated.

In conclusion, our study of outdoor workers on ski resorts reveals a lower prevalence of skin cancer than other groups of outdoor workers. Their sun protection practices are good although the frequency of application of the sunscreen would improve. 

## Figures and Tables

**Table 1:** Photoprotection habits of the study population.

Habits	*N*	%
*When they use photoprotection*		
(1) Always	106	48.4%
(2) Sunny & cloudy	47	21.5%
(3) Only sunny days	62	28.3%
(4) Unknown	4	1.8%

*Use sunscreen >25*		
(1) Always	148	67.6%
(2) Sometimes	44	20%
(3) Never	17	7.8%
(4) Unknown	10	4.6%

*Use of hat/helmet*		
(1) Always	165	75.2%
(2) Sometimes	40	18.2%
(3) Never	14	6.3%
(4) Unknown	1	0.3%

*Use of glasses*		
(1) Always	213	97.3%
(2) Sometimes	4	1.8%
(3) Never	2	0.9%

*Use of shadow*		
(1) Always	37	16.9%
(2) Sometimes	78	35.6%
(3) Never	104	47.5%

**Table 2:** Characteristics of the study population and associations with the presence of actinic keratosis lesions and photoaging grade.

	Presence of AK lesions		*p*	Photoaging grade				
	Yes	No		Grade 1	Grade 2	Grade 3	Grade 4	*p*
				*n* (%)	*n* (%)	*n* (%)	*n* (%)	
Mean no. of years working in ski resorts (SD, range)	29.1(9.7, 10–50)	18.3(12, 1–45)	**<0.001**	11.19 (9.23)	14.08 (9.43)	27.17 (12.0)	31.95 (11.32)	**<0.001**

Mean age in years (SD, range)	51.7 (7.4, 35–66)	42.5 (11.3, 19–66)	**<0.000**	35.14 (10.98)	39.27 (9.77)	49.87 (8.17)	54.65 (6.34)	**<0.001**

*Sex*			**0.001**					0.632
(1) Male	32 (100%)	145 (77.5%)		28 (75.7%)	67 (80.7%)	64 (81%)	18 (90%)	
(2) Female	0 (0%)	42 (22.5%)		9 (24.3%)	16 (19.3%)	15 (19%)	2 (10%)	

*Phototype*			0.876					**0.002**
1	5 (15.6%)	28 (15%)		2 (5.4%)	19 (22.9%)	11 (13.9%)	1 (5%)	
2	12 (37.5%)	64 (34.2%)		13 (35.1%)	33 (39.8%)	26 (32.9%)	4 (20%)	
3	6 (18.8%)	58 (31%)		14 (37.8%)	17 (20.5%)	29 (36.7%)	4 (20 %)	
4	8 (25%)	30 (16%)		6 (16.2%)	11 (13.3%)	13 (16.5%)	8 (40%)	
5	1 (3.1%)	7 (3.7%)		0 (0)	3 (3.6%)	0	3 (15%)	

*Hair colour*			**0.039**					0.989
(1) L-brown	9 (28.1%)	43 (23%)		0	1 (1.2%)	1 (1.3%)	0	
(2) D-brown	14 (43.8%)	75 (40.1%)		3 (8.1%)	10 (12%)	9 (11.3%)	2 (10%)	
(3) Black	2 (6.3%)	50 (26.7%)		7 (18.9%)	20 (24.1%)	19 (24.1%)	6 (30%)	
(4) Redhead	1 (3.1%)	1 (0.5%)		16 (43.2%)	31 (37.3%)	33 (41.8%)	9 (45%)	
(5) Blond	6 (18.8%)	18 (9.6%)		11 (29.7%)	21 (25.3%)	17 (21.5%)	3 (15%)	

*Eye colour*			0.207					0.908
(1) Blue	7 (21.9%)	20 (10.7%)		3 (8.1%)	11 (13.3%)	10 (12.7%)	3 (11.1%)	
(2) Green	6 (18.8%)	24 (12.8%)		5 (13.5%)	13 (15.7%)	9 (11.4%)	3 (15%)	
(3) D-green	5 (15.6%)	25 (13.4%)		2 (5.4%)	12 (14.5%)	13 (16.5%)	3 (15%)	
(4) L-Brown	8 (25%)	51 (27.3%)		10 (27%)	21 (25.3%)	22 (27.8%)	6 (30%)	
(5) D-Brown	6 (18.8%)	67 (35.8%)		17 (45.9%)	26 (31.3%)	25 (31.6%)	5 (25%)	

*History of skin cancer*			0.055					0.355
(1) BCC	2 (6.3%)	2 (1.1%)		0	1 (1.2%)	2 (2.5%)	1 (5%)	
(2) AK	2 (6.3%)	1 (0.5%)		1 (2.7%)	0	2 (2.5%)	0	
(3) Other	0 (0%)	1 (0.5%)		1 (2.7%)	0	0	0	
(4) None	28 (87.4%)	183 (97.9%)		25 (94.6%)	82 (98,8%)	75 (94.9%)	19 (95%)	

*Freckles on back*			**0.021**					**<0.001**
(1) Many	7 (21.9%)	10 (5.3%)		24 (64.9%)	38 (45.8%)	18 (22.8%)	8 (40%)	
(2) Moderate	5 (15.6%)	25 (13.4%)		4 (10.8%)	16 (19.3%)	33 (41.7%)	4 (20%)	
(3) Few	11 (34.4%)	46 (24.6%)		2 (5.4%)	9 (10.8%)	17 (21.5%)	2 (10%)	
(4) None	7 (21.9%)	81 (43.3%)		3 (8.1%)	3 (3.6%)	7 (8.9%)	4 (20%)	
(5) Unknown	2 (6.3%)	25 (13.3%)		4 (10.8%)	17 (20.5%)	4 (5.1%)	2 (10%)	

*Nevi on head*			0.552					0.386
(1) 0	13 (40.6%)	71 (38%)		12 (32.4%)	26 (31.3%)	36 (45.6%)	10 (50%)	
(2) 1–5	15 (46.8%)	102 (54.5%)		21 (56.8%)	49 (59%)	39 (49.4%)	8 (40%)	
(3) >5	4 (12.5%)	14 (7.5%)		4 (10.8%)	8 (9.6%)	4 (5.1%)	2 (10%)	

*Nevi on back*			**0.039**					0.356
(1) 0–10	12 (37.5%)	112 (59.9%)		23 (62.3%)	44 (53%)	47 (59.5%)	10 (50%)	
(2) 11–25	10 (31.3%)	50 (26.7%)		12 (32.4%)	26 (31.3%)	15 (19%)	7 (35%)	
(3) 26–50	9 (28.1%)	19 (10.2%)		2 (5.4%)	9 (10.8%)	14 (17.7%)	3 (15%)	
(3) >50	1 (3.1%)	6 (3.2%)		0	4 (4.8%)	3 (3.8%)	0	

*Nevi on trunk*			0.510					0.649
(1) 0–10	20 (62.5%)	136 (72.7%)		31(83.8%)	54 (65.1%)	57 (72.2%)	14 (70%)	
(2) 11–25	9 (28.1%)	42 (22.5%)		5 (13.5%)	24 (28.9%)	17 (21.5%)	5 (25%)	
(3) 26–50	3 (9.4%)	8 (4.3%)		1 (2.7%)	4 (4.8%)	5 (6.3%)	1 (5%)	
(4) >50	0 (0%)	1 (0.5%)		0 (0%)	1 (1.2%)	0 (0%)	0 (0%)	

*Nevi on upper extremities*			0.686					0.478
(1) 0–10	20 (62.5%)	134 (71.7%)		30 (81.1%)	52 (62.7%)	58 (73.4%)	14 (70%)	
(2) 11–25	10 (31.3%)	44 (23.5%)		7 (18.9%)	26 (31.3%)	17 (21.5%)	4 (20%)	
(3) 26–50	2 (6.3%)	8 (4.3%)		0 (0%)	4 (4.8%)	4 (5.1%)	2 (10%)	
(4) >50	0 (0%)	1 (0.5%)		0 (0%)	1 (1.2%)	0 (0%)	0 (0%)	

*Nevi on lower extremities*			0.52					0.074
(1) 0–10	30 (93.8%)	173 (92.5%)		36 (97.3%)	79 (95.2%)	68 (86.1%)	20 (100%)	
(2) 11–25	2 (6.3%)	10 (5.3%)		1 (2.7%)	2 (2.4%)	9 (11.4%)	0 (0%)	
(3) 26–50	0 (0%)	4 (2.1%)		0 (0%)	2 (2.4%)	2 (2.5%)	0 (0%)	

*Photoaging grade*			**<0.001**					0.177
1	1 (3.1%)	36 (19.3%)						
2	7 (21.9%)	76 (40.6%)						
3	17 (53.1%)	62 (33.2%)						
4	7 (21.9%)	13 (7%)						

L-brown, light brown; D-brown, dark brown; BCC: basal cell carcinoma; AK: actinic keratosis. In bold those data with statistical significance.

**Table 3:** Photoprotective measures and association with the presence of actinic keratosis lesions.

Photoprotective measures	Presence of AK		*p*
	Yes	No	
*Sunscreen SPF >25*			0.267
(1) Always	20 (62.5%)	138 (73.8%)	
(2) Sometimes	9 (28.1%)	35 (18.7%)	
(3) Never	3 (9.4%)	14 (7.5%)	

*Hat/helmet*			0.501
(1) Always	23 (71.9%)	142 (75.9%)	
(2) Sometimes	6 (18.8%)	34 (18.2%)	
(3) Never	3 (9.4%)	11 (5.9%)	

*Sunglasses*			0.131
(1) Always	30 (93.8%)	183 (97.9%)	
(2) Sometimes	1 (3.1%)	3 (1.6%)	
(3) Never	1 (3.1%)	1 (0.5%)	

*Shade*			**0.005**
(1) Always	10 (31.3%)	27 (14.4%)	
(2) Sometimes	13 (40.6%)	65 (34.8%)	
(3) Never	9 (28.1%)	95 (50.8%)	

Bold letters mean statistical significance.

**Table 4:** Association between colorimetry and presence of actinic keratosis lesions.

	Presence of AK		*p*
	Yes	No	
Erythema face (Mean (SD))	20.21 (4.01)	20.33 (3.23)	0.852
Erythema armpit (Mean (SD))	10.65 (3.55)	10.89 (3.40)	0.717
Melanin face (Mean (SD))	41.77 (4.26)	42.61 (5.73)	0.333
Melanin armpit (Mean (SD))	35.42 (5.73)	33.67 (3.43)	0.105
Erythema: face vs. armpit difference (Mean (SD))	9.53 (6.55)	10.71 (9.27)	0.402
Melanin: face vs. armpit difference (Mean (SD))	6.40 (7.73)	9.75 (9.26)	0.060

## Data Availability

The data used to support the findings of this study are included within the article and can be used by other researchers once the article will be published.

## Supplementary Materials

Supplementary Materials Characteristics of the study population.
