# Supplementary material for: Skin Cancer Prevalence in Outdoor Workers of Ski Resorts
Source: J Skin Cancer. 2020 Jan 28;2020:8128717. doi: 10.1155/2020/8128717 (PMC7097757; doi:10.1155/2020/8128717)
Supplement: Supplementary Materials — Characteristics of the study population. [file 8128717.f1.docx]

Table Suppl. Characteristics of the study population.

| Characteristics | N | % |  |
| --- | --- | --- | --- |
| Age (average, SD, range) | 43.8 (11.31)  (19-66) |  |  |
| Years working in ski resorts (mean, SD, range) | 20.21 (12.92)  (1-50) |  |  |
| Sex  - Men  - Women | 177  42 | 80.8  19.2 |  |
| Phototype  1  2  3  4  5 | 33  76  64  38  8 | 15.1  34.7  29.2  17.4  3.6 |  |
| Hair colour  - Light brown  - Dark brown  - Black  - Redhead  - Blond | 52  89  52  2  24 | 23.7  40.7  23.7  0.9  11 |  |
| Eye colour  - Blue  - Green  - Dark green  - Light Brown  - Dark Brown | 27  30  30  59  73 | 12.3  13.7  13.7  26.9  33.4 |  |
| History of skin cancer  - Basal cell carcinoma  - Actinic keratosis  - Other  - None | 4  3  1  211 | 1.8  0.5  0.5  96.2 |  |
| Freckles on the back  - Many  - Moderate  - Few  - None  - Unknown | 17  30  57  88  27 | 7.7  13.7  26  40.3  12.3 |  |
| Nevi on head  - 0  - 1-5  - >5 | 84  117  18 | 38.4  53.4  8.2 |  |
| Nevi on trunk  - 0-10  - 11-25  - 26-50  - >50 | 156  51  11  1 | 71.2  23.3  5  0.5 |  |
| Nevi on lower extremities  - 0-10  - 11-25  - 26-50 | 203  12  4 | 92.7  5.5  1.8 |  |
| Nevi on back  - 0-10  - 11-25  - 26-50  - >50 | 124  60  28  7 | 56.6  27.4  12.8  3.2 |  |
| Nevi on upper extremities  - 0-10  - 11-25  - 26-50 | 154  54  10  1 | 70.3  24.7  4.6  0.4 |  |
| Photoaging grade  I  II  III  IV | 37  83  79  20 | 16.9  38.0  36.1  9.1 |  |
| Nº number of sunburns per season (mean, SD)  0  1  2  3  4  5  8  Unknown | 0,79 (1,30)  124  53  13  12  4  6  1  6 | 56.6  24.2  5.9  5.5  1.8  2.7  0.6  2.7 |  |
